# The effect of information overload and perceived risk on tourists’ intention to travel in the post-COVID-19 pandemic

**DOI:** 10.3389/fpsyg.2022.1000541

**Published:** 2022-10-25

**Authors:** Hong Wu, Qi Cao, Jia-Min Mao, Hui-Ling Hu

**Affiliations:** ^1^School of Tourism, Hainan University, Haikou, China; ^2^School of Humanities and Social Science, Hong Kong University of Science and Technology, Kowloon, Hong Kong SAR, China; ^3^College of Humanities, Hainan University, Haikou, China; ^4^Quantitative Analysis and Research Association, Kaohsiung, Taiwan

**Keywords:** information overload, perceived risk, tourism industry, theory of reasoned action (TRA), post-COVID-19 pandemic

## Abstract

Since the COVID-19 pandemic, the tourism economy has been seriously affected. China has implemented a direct traveling management mechanism and recovered from the pandemic faster than the rest of the world. However, the COVID-19 situation is complicated and uncontrollable because of the available unclear information including difficult medical terminologies. This study attempts to find the determinants of the travel intention of China’s tourists in the post-COVID-19 epidemic. Along with information overload and perception risk, an expanded research model of the Theory of Planned Behavior (TPB) was employed to propose the theoretical framework of this study. A survey was conducted among 518 tourists who spend their holiday in Hainan, which is a popular tourist destination in China. The empirical results show that information overload positively and significantly impacted perceived risk. Furthermore, perceived risk negatively affects the intention to travel. Perceived risk also negatively affected the attitude toward traveling. However, response self-efficacy did not have a significant effect on the intention to travel. Finally, based on the analysis results, this study proposes relevant research contributions and practical recommendations with management implications for the travel industries.

## Introduction

Currently, various industries faced issues during the COVID-19 pandemic. However, tourism is deemed to be one of the most seriously impacted industries. The subsequent changing behaviors of tourists in the post-COVID landscape are worthy of attention to many researchers. De Vos (2020) discussed the potential effects of avoiding social contact and reduced interaction under measures to combat the spread of COVID-19. Furthermore, social distancing and restricted traveling result in social isolation and limited activity. In addition, Brooks et al. (2020) illustrated the psychological impact of quarantine and concluded that it has several negative psychological effects. The study recommended defining a clear rationale and information for quarantine and ensuring sufficient supplies. Neuburger and Egger (2020) determined the relationship between the perception of travel risk and the travel behavior of travelers during the COVID-19 pandemic.

Moreover, there is adequate information available regarding COVID-19, including views on virus mutation and vaccination development on the internet. Mutant viruses, such as Alpha, Beta, Gamma, and Delta, have been discovered among various COVID-19 patients (Jason Gale, 2021). The COVID-19 pandemic has brought a series of new challenges in terms of information overload (Rathore and Farooq, 2020). The COVID-19 pandemic has caused disorder in various areas. For instance, vaccines that were developed in many countries are being used but the unknown nature of the pandemic means that it is difficult to predict future trends. Additionally, the information about the pandemic is also complicated and diverse so it is often contradictory. Also, there is an overloaded and chaotic information flow for the general population. This negates the effect of measures to control the spread of COVID-19 infections (Mohammed et al., 2021).

Hainan is the southernmost island in China and has been a popular tourist destination in China for many years. China tourists visit Hainan for its warm climate, beaches, and palm trees. In recent years, it has become an international business conference and exhibition center. In the post-COVID-19 era, Hainan’s tourism industry has also been significantly affected.

After the COVID-19 pandemic, on January 24, 2020, the government of Hainan began a community wellbeing emergency response and encouraged the scale response of community wellbeing emergencies on January 25, 2020. This implies that past January 24th, 2020, the inner movement of individuals was significantly contained, and Hainan nearly did not have any tourists for a section of the period in January. However, owing to efficient community supervision and health care, the pandemic in China was effectively controlled, and the people’s movement started to continue in an organized way (Liu et al., 2021).

This study surveys travelers of Hainan in the post-COVID-19 era to determine the effect of information overload, perceived risk, attitude, and response efficacy on intention to travel. The research study also employs subjective norms as a moderating variable to determine the effect of public opinions on the relationship between perceived risk and intention to travel. Furthermore, the theoretical framework of this study was built on theories and concepts related to consumer behavior, information overload, perception risk, and an expanded research model of the Theory of Planned Behavior (TPB). The results of the study examine the effect of information overload on the intention to travel and the importance of correct and useful information in the post-COVID-19 era.

## Theoretical background

### Information overload

Miller (1956) first proposed the concept of information overload, which means that at any given time, through psychological research, the processing power of humans is very limited. The maximum amount of information that can consciously be processed is about seven units. Consumer decision-making was later studied by Jacoby et al. (1974) to define the theory of information overload. Furthermore, Matthes et al. (2020) noted that overburdening with information, permanent online interaction with other users, the perception of permanent observation, and demand for reactions result in information overload for mobile social networking sites (SNS) use.

Information overload refers to a state whereby a person believes that there is an imbalance between environmental needs and the available resources that can be used to respond to these needs (Eppler and Mengis, 2004). Other academic disciplines, such as cognitive psychology, focus on the acute phenomenon of information overload, which affects recall, judgment, and decision-making (Bargh and Thein, 1985). Fu et al. (2020) noted that overloading describes a person’s subjective perception and evaluation of the amount of information, persons, or objects that exceed the processing capacity. In an era of information and communication technologies, overload is a key factor in providing negative results (Lee et al., 2016). Various devices and applications can increase personal perceptual overload (Yin et al., 2018).

At the beginning of the COVID-19 epidemic, inaccurate information and information overload caused consumers to engage in panic buying (Herjanto et al., 2021). The complex development of the pandemic and emotional news reports resulted in information overload and there is a lack of experience with decision-making in a situation of information overload (Farooq et al., 2021).

### Perceived risk

Perceived risks that are related to health, well-being, and safety create a preference for specific travel choices (Ozbilen et al., 2021). Traveling cannot be experienced in advance and it is impossible to know the risks that pertain to travel. Perceived risk is an important factor for predicting travel behavior because it explains the important factors in the choice of a travel destination (Zilker et al., 2020).

Previous research has also demonstrated that the perceptions of service quality, value, destination image, risk, subjective norm, and behavioral control are critical factors for attitude and intention to travel (Abbasi et al., 2021). Perceived risk is also proven to negatively impact participation in out-of-home activities and the frequency of trips (Parady et al., 2020).

### Theory of planned behavior

Under the basic idea of the theory of reasoned action, Ajzen (1991) proposed TPB to determine the mechanism for behavioral intention, which explains human behaviors. The TPB assumes that individuals are rational and evaluate alternatives and form intentions before taking actions. The common factors of behavioral intention include attitude, subjective norm, and perceived behavior control. Attitude is a person’s general perception of specific behavior, which can be positive or negative. Subjective norms determine the way the opinions of other individuals affect the person in terms of a particular behavior. Perceptual behavior control is a factor that facilitates or hinders behavior. The TPB model states that the three pre-variables of behavior attitude, subjective norm, and perceived behavioral control positively affect behavior intentions (Ajzen, 1991).

### Response efficacy

Bandura’s (1998) study defined self-efficacy as the ability of an individual to believe in an ability to organize and perform a specific achievement (Bandura, 1998). This is the perceived behavior control construct for the TPB. Ajzen’s (2002) study noted that self-efficacy and perceived behavior control are two mutually interchangeable aspects. Ajzen divided the perceived behavioral control construct into two components: self-efficacy and controllability. Self-efficacy pertains to the degree of difficulty in executing a behavior. Some studies show that self-efficacy has a direct effect on intention (Cooke et al., 2016).

Self-efficacy is defined as strong self-confidence in specific abilities, which reduces stress and anxiety. Response efficacy defines how individuals perceive their confidence in a response to a specific situation. Response efficacy affects the intention to secure information (Menard et al., 2017).

## Hypotheses development

### Information overload and perceived risk

When insufficient useful information is provided by a product or service, perceived risk increases. In a situation of information overload, doubts and uncertainty prevail and the efficiency and quality of decision-making are reduced (Castañeda et al., 2020). In addition, the information about COVID-19 can be overwhelming so the causal relationship between information overload and perceived risk during the COVID-19 pandemic is defined in terms of the following hypothesis:

**H1**: Information overload has a positive impact on the perceived risk of travelers in the post-COVID-19 era.

### Perceived risk, traveling attitude, and intention to travel

The TPB explains the behavioral willingness mechanism. After COVID-19, many empirical studies of travel behavior reference the TPB theory and its mechanism. The study by Rahmafitria et al. (2021) uses knowledge, social attention, and implementation risk in the COVID-19 pandemic era to expand the TPB theory in the field of tourism. The study collected questionnaires in Indonesia and found that subjective norms have a greater effect than attitudes. Furthermore, the effect of behavioral control was found to be significant. Knowledge, perceived risks, and social concerns also significantly affect willingness to travel.

Tasci and Sönmez (2019) noted that an individual’s attitude is significantly affected by perceptions. Whenever there is a decision to travel, the perceived risk is the factor that tourists take into consideration before traveling (Beirman, 2006; Rittichainuwat et al., 2018). Furthermore, uncertainty, chaotic information, and the development of COVID-19 might also confuse travel behavior.

Previous studies determined the effect of information overload on perceived risk. More contradictory information results in unproductive attitudes (Cavlek, 2002; Rahmafitria et al., 2021). This study proposes that the construct of perceived risk significantly affects traveling attitude and intention in the COVID-19 era. The TPB also shows that traveling attitude has a direct effect on intention. Hence, the following hypotheses are postulated.

**H2**: Perceived risk has a negative impact on traveling attitude in the post-COVID-19 era.

**H3**: Perceived risk has a negative impact on intention to travel in the post-COVID-19 era.

**H4**: Traveling attitude has a positive impact on intention to travel in the post-COVID-19 era.

### Response efficacy and intention to travel

In the context of global pandemic research, response efficacy accurately predicts individuals’ adaptive behaviors, such as vaccination behaviors (Davis et al., 2021). This study uses response efficiency to determine individuals’ confidence in their response to difficulties during the COVID-19 pandemic while traveling. The TPB states that traveling response efficacy directly impacts the intention to travel. Hence, the following hypotheses can be postulated.

**H5**: Response efficacy has a positive impact on intention to travel in the post-COVID-19 era.

### Mediating and moderating effects

This study determines the way information overload and perceived risk affect tourists’ intention to travel since the COVID-19 pandemic. The greater the information overload, the greater the perceived risk of traveling. Furthermore, the perceived risk decreases the intention to travel. Therefore, the mediating effect of the perceived risk on the intention to travel is tested using the following hypothesis:

H6: Perceived risk has a mediating effect between information overload and intention to travel in the post-COVID-19 era.

During the COVID-19 pandemic, government travel rules required nucleic acid testing, wearing of masks, isolation in hotels, and home isolation for travelers. In this way, the opinions of others might affect the travel decisions of potential tourists. Furthermore, research conducted by Schepers and Wetzels (2007) analyzed subjective norms, the technology acceptance model (TAM), and, behavioral intention. The research also included moderating effects of an individual-associated variable, a technology-associated variable, and a dependent variable. Findings suggested a substantial impact of the subjective norms with perceived usefulness and intention. Moreover, moderating impacts were discovered for each designated variable (Schepers and Wetzels, 2007). This study proposes the use of a subjective norm as a moderating variable to determine the significance related to the opinions of others. Hence, the following hypothesis can be postulated.

**H7**: A subjective norm has a moderating effect between perceived risk and intention to travel in the post-COVID-19 era.

**FIGURE 1:**
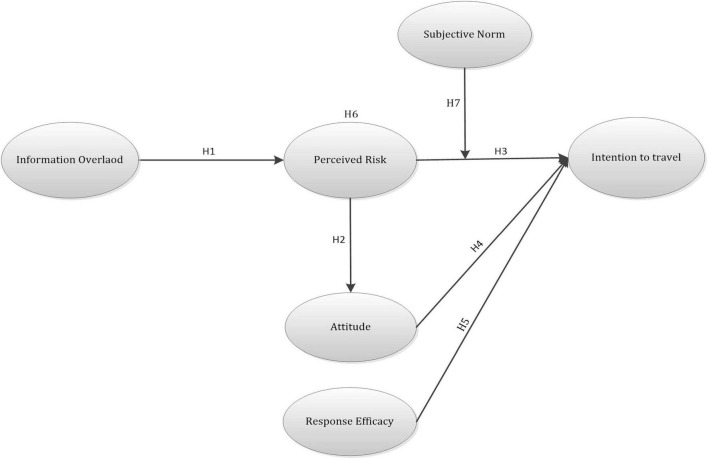
Research framework.

According to the above hypothesis development, the research framework is proposed in Figure 1.

## Methodology

### Data collection

To study the intention to travel after the COVID-19 epidemic, this study surveyed individuals over the age of 20. Self-administered questionnaires and online electronic surveys were used for data collection. Questionnaires were distributed in major resort hotels, stations, and airports to collect data in Hainan from 1 May 2021 to 30 May 2021. A total of 518 effective questionnaires were collected.

### Measurement instrument

The research variables for this study include the background information for tourists and research variables. To verify the concepts of the research variables, the operational definitions of the following variables are explained as follows. The basic personal data is measured in the first part of the questionnaire and includes seven items: gender, age, marital status, occupation, home environment, and location. All research variables are measured using a 7-point Likert scale, for which one means strongly disagree and seven means strongly agree. The survey instructions for each construct are shown as follows.

This study refers to the definition of information overload in the study by Chen et al. (2009) to determine the effect of information overload on decision-making for e-retail consumers. The results of that study show that an excess of information leads to a high degree of information overload. Four items are excessive information, processing pressure, difficulty in understanding information, and difficulty in obtaining information sources.

The variable of perceived risk variable includes a total of six items. The items were adopted and modified from a study by Abrahão et al. (2016) that compared the mobile phone customers for a telecommunications company in southeastern Brazil and determined the relationship between performance expectations, effort expectations, social influence, and perceived risk.

The items for the traveler’s attitude were adopted and modified from a study conducted by Paul et al. (2016), which defined the items for consumer attitudes to green products. This study showed that attitude and behavior control perception for green products accurately predict purchase intentions and subjective norms do not. The Cronbach’s α value for the attitude construct is 0.897. This study also employs some items from Mehrad and Mohammadi’s (2017) study, which determined the effect of Iran’s mobile banking reputation on the adoption rate. The results showed that word-of-mouth is the main factor affecting users’ attitudes toward mobile banking. In terms of the subjective norm, this study uses the items suggested by Taufique and Vaithianathan’s (2018) study. There are five items for subjective norm constructs.

In terms of the items for the intention to travel construct, the items suggested by Jang et al.’s (2015) study are used. Their study determined the relationship between precedents in terms of environmental awareness behaviors, perceived consumer effectiveness, environmental concerns (EC), and attitude (AT) associated with the TPB to determine consumer behavioral intentions (BI).

In terms of response efficacy items, this study uses those for the study by Rimal and Real (2003), which defines response efficacy as a subconstruct of the efficacy belief. There are four items for the response efficacy. The items are modified to suit this study and are available in the appendix part of the study as Table 1.

**TABLE 1:** Measurement items and sources.

Constructs	Items	References
Information overload	(1) Since the COVID-19 pandemic, I feel that there is too much travel information.	Chen et al., 2009
	(2) Since the new crown epidemic, there has been too much travel information, and I feel pressured to deal with it.	
	(3) Since the COVID-19 pandemic, I feel it is too difficult to understand the information that I need.	
	(4) Since the COVID-19 pandemic, I don’t know where to find the information I need.	
Perceived risk	(1) Since the COVID-19 pandemic, I have not felt safe when traveling.	Abrahão et al., 2016
	(2) Since the COVID-19 pandemic, I have not felt well protected.	
	(3) Since the COVID-19 pandemic, there is a high possibility of problems during travel.	
	(4) Since the COVID-19 pandemic, traveling has been dangerous for me.	
	(5) Since the COVID-19 pandemic, travel involves a lot of risks.	
	(6) Since the new crown epidemic, tourism increases uncertainty.	
Attitude	(1) I like traveling.	Paul et al., 2016; Mehrad and Mohammadi, 2017
	(2) Traveling is a good idea.	
	(3) I have a good attitude toward traveling.	
	(4) Traveling fits my lifestyle.	
	(5) Travel makes life more interesting.	
Subjective norm	(1) After the COVID-19 pandemic, my friend approved of my traveling.	Taufique and Vaithianathan, 2018
	(2) After the COVID-19 pandemic, my neighbor approved of my traveling.	
	(3) After the COVID-19 pandemic, my colleague agreed with me to travel.	
	(4) After the new crown epidemic, my family approved of my traveling.	
	(5) After the COVID-19 pandemic, my friends agreed with me to travel.	
Intention to travel	(1) I plan to travel to a resort.	Jang et al., 2015
	(2) I would like to travel to a resort.	
	(3) I am willing to spend time and money to travel to a resort.	
	(4) I have traveled a lot recently.	
	(5) I will encourage others to travel.	
Response efficacy	(1) There are many things I can do to make sure that I remain free of COVID-19.	Rimal and Real, 2003
	(2) I have the necessary skill to enact good COVID-19 measures to keep me free of COVID-19 during trips.	
	(3) I have the necessary knowledge to enact good COVID-19 measures to keep me free of COVID-19 during trips	
	(4) I have the necessary competencies to enact good COVID-19 measures to keep me free of COVID-19 during the trips.	

## Results

### Sample structure

This study collected a total of 518 valid questionnaires in Hainan, at airports, restaurants, hostels, and shopping malls that are frequently visited by tourists, using a convenience sampling method. The sample distribution is shown in Table 2. A total of 304 female respondents accounted for 58.7% of the population and 214 male respondents accounted for 41.3%. In terms of age, 302 people aged between 31 and 40 accounted for 58.3%, and 80 people aged between 20 and 30 accounted for 15.4%. In terms of marriage, 333 were unmarried, which accounted for 64.29%, and 179 were married, which accounted for 34.56%. A total of 186 people took more than three trips per year, which accounted for 35.9%, and 155 people took two trips per year, which accounted for 29.9%. In terms of occupation, 193 students accounted for 37.3% and 102 corporate employees accounted for 19.7%. In terms of educational background, 354 university graduates accounted for 68.3%.

**TABLE 2:** Sample structure.

	Value label	Frequency	Percent
**Gender**	Male	214	41.3
	Female	304	58.7
**Age**	20–30 years old	80	15.4
	31–40 years old	302	58.3
	41–50 years old	78	15.1
	51–60 years old	35	6.8
	61–70 years old	13	2.5
	71 years old or above	10	1.9
**Marital status**	Married	179	34.56
	Unmarried	333	64.29
	Other	6	1.16
**Trip per year**	1	110	21.2
	2	155	29.9
	3	67	12.9
	Above 3	186	35.9
**Occupation**	Government worker	24	4.6
	Institutional personnel	51	9.8
	Corporate employees	102	19.7
	Business person	31	6.0
	Student	193	37.3
	Freelance	60	11.6
	Other	57	11.0
**Education Level**	Junior high school and below	7	1.4
	High school	29	5.6
	College	71	13.7
	University	354	68.3
	Graduate school	57	11.0

### Descriptive statistics analysis

The results for the descriptive statistics showed that the average number of 2.900 for INO4 was the lowest, with 4.760 for ATT3 and ATT4 being the highest. For this study, the value for Kurtosis was between –1.090 and –0.100. Additionally, for Skewness the values were between –0.670 and 0.620, as shown in Table 3. The empirical rule of Kline (2015) states that if the absolute value for skewness is less than or equal to 2 and the absolute value for Kurtosis is less than 7, then the variable conforms to the univariate normality. For this study, the absolute values of skewness and kurtosis are less than 2.

**TABLE 3:** Mean and standard deviation for items.

Variable	*N*	Mean	Standard deviation	Skewness	Kurtosis
INO1	518	3.650	1.470	0.170	–0.530
INO2	518	3.160	1.430	0.490	–0.270
INO3	518	3.050	1.480	0.550	–0.340
INO4	518	2.900	1.540	0.620	–0.450
PR1	518	4.180	1.700	–0.210	–1.090
PR2	518	3.870	1.610	0.040	–0.960
PR3	518	3.970	1.640	0.020	–0.910
PR4	518	3.950	1.620	0.070	–0.930
PR5	518	4.030	1.630	–0.030	–0.930
PR6	518	4.610	1.580	–0.460	–0.710
IT1	518	4.190	1.680	–0.270	–0.880
IT2	518	4.290	1.700	–0.390	–0.850
IT3	518	4.130	1.690	–0.300	–0.880
IT4	518	3.310	1.650	0.350	–0.760
IT5	518	3.070	1.600	0.480	–0.680
ATT1	518	4.640	1.660	–0.560	–0.470
ATT2	518	4.320	1.660	–0.310	–0.770
ATT3	518	4.760	1.540	–0.670	–0.100
ATT4	518	4.760	1.630	–0.660	–0.340
ATT5	518	4.690	1.640	–0.480	–0.530
SUN1	518	3.680	1.630	0.010	–0.830
SUN2	518	3.320	1.460	0.110	–0.590
SUN3	518	3.540	1.570	0.000	–0.770
SUN4	518	3.300	1.660	0.210	–0.950
REE1	518	4.680	1.490	–0.490	–0.380
REE2	518	4.510	1.520	–0.320	–0.560
REE3	518	4.750	1.460	–0.490	–0.270
REE4	518	4.540	1.480	–0.300	–0.610

INO, information overload; PR, perceived risk; IT, intention to travel; ATT, attitude; SUN, subjective norm; REE, response efficacy.

### Confirmatory factor analysis

This study uses a two-stage approach to verify the proposed model using structural equation modeling (SEM), as suggested by Anderson and Gerbing (1988). Stage 1 was associated with analyzing the variables’ validity and reliability. Confirmatory factor analysis (CFA) and Fornell and Larcker criterion were used in this stage. The second stage determined the effect of the path on the structural model and its significance (Anderson and Gerbing, 1988; Hulland, 1999). Mediation and moderation analysis were also conducted in Stage 2. Furthermore, to deal with the complex proposed research model, past studies have considered PLS as a suitable choice for the analysis of this study (Chin and Newsted, 1999; Zhao and Khan, 2021).

Table 4 shows the standardized factor loadings for scales from 0.560 to 0.941, which was a reasonable range. The composite reliability for each dimension was 0.818–0.937, exceeding 0.7, as recommended by Nunnally (1994) and Hair et al. (2010), so all dimensions exhibit internal consistency. The lowest value for average variance extracted (AVE) was 0.607, which exceeded the value of 0.5 suggested by Fornell and Larcker (1981) and Hair et al. (1998), so all dimensions exhibit convergent validity.

**TABLE 4:** Reliability and convergent validity.

Dimension	Scale	Standardized factor loading	*Z*-value	CR	AVE
INO	INO2	0.560		0.818	0.607
	INO3	0.866	12.731		
	INO4	0.871	12.747		
PR	PR1	0.605		0.910	0.634
	PR2	0.731	13.824		
	PR3	0.875	15.264		
	PR4	0.915	15.493		
	PR5	0.897	15.456		
	PR6	0.704	13.277		
IT	IT1	0.928		0.923	0.711
	IT2	0.941	40.891		
	IT3	0.941	40.247		
	IT4	0.687	19.586		
	IT5	0.672	18.859		
ATT	ATT1	0.876		0.937	0.749
	ATT2	0.873	28.058		
	ATT3	0.869	27.122		
	ATT4	0.888	28.149		
	ATT5	0.819	24.357		
REE	REE1	0.840		0.937	0.789
	REE2	0.871	25.660		
	REE3	0.933	28.479		
	REE4	0.907	26.896		

CR, composite reliability; AVE, average variance extracted; INO, information overload; PR, perceived risk; IT, intention to travel; ATT, attitude; REE, response efficacy.

To confirm the discriminant validity, the square root of the AVE for a dimension is compared with the correlation. As shown in Table 5, the square root of the AVE for all five dimensions of this study is greater than the correlation coefficient, so the data of this study has discriminant validity (Fornell and Larcker, 1981).

**TABLE 5:** Correlations and discriminant validity.

	AVE	INO	PR	IT	ATT	REE
INO	0.607	**0.779**				
PR	0.634	0.364	**0.796**			
IT	0.711	–0.104	–0.286	**0.843**		
ATT	0.749	–0.052	–0.142	0.747	**0.865**	
REE	0.789	0.001	0.000	0.067	0.000	**0.888**

The items on the diagonal in bold font are the square roots of the AVE; off-diagonal elements are the correlation estimates.

Large-sample studies usually have a *p*-value of less than 0.05, so the SEM model fitting is easily affected by a bad result (Schumacker and Lomax, 2004). Therefore, the model and sample for a quantitative study must be matched and verified using many different methods. This study uses eight methods to test the model fit: Normed Chi-square, CFI, GFI, AGFI, TLI, and RMSEA (as shown in Table 6). All of the models fit criteria signified fit according to the suggested standards.

**TABLE 6:** Model fit.

Model fit	Criteria	Model fit of the proposed model
Normed Chi-square (χ^2^/DF)	χ^2^/DF < 5	3.704
CFI	>0.9	0.922
GFI	>0.9	0.921
AGFI	>0.9	0.911
TLI (NNFI)	>0.9	0.912
RMSEA	<0.08	0.072

### Structural model analysis

**FIGURE 2:**
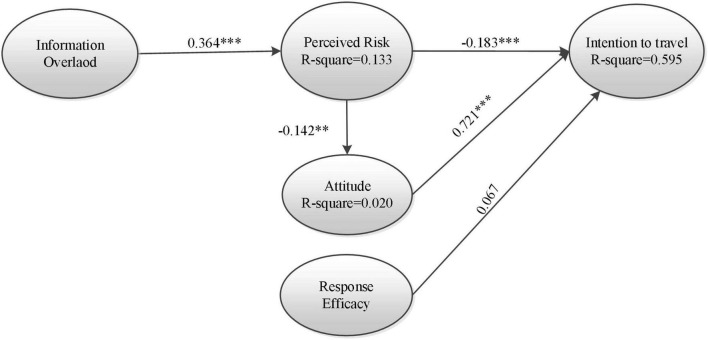
Results of the structural model. **p < 0.01, ***p < 0.001.

Table 7 and Figure 2 show the results for path coefficients. According to the results, information overload significantly impacted perceived risk (β = 0.364, *p* < 0.001). Moreover, the intention to travel was significantly impacted by perceived risk (β =–0.183, *p* < 0.001) and attitude (β = 0.721, *p* < 0.001); however, it was not a significantly impacted response efficacy (β = 0.067). Furthermore, perceived risk also significantly impacted attitude (β =–0.142, *p* = 0.003). These results support the validity of the research model. A total of 13.3% of perceived risk is explained by the information overload construct, and 59.5% of intention to travel is explained by perceived risk, attitude, and response efficacy constructs. In addition, 2% of attitude is explained by the perceived risk construct.

**TABLE 7:** Research hypotheses testing.

Endogenous variables	Exogenous variables	Standardized path coefficient (β)	Regression weight	Stand error	*Z*-value	*P*-value
PR	INO	0.364	0.468	0.072	6.468	0.000
IT	PR	–0.183	–0.273	0.051	–5.399	0.000
	ATT	0.721	0.761	0.042	17.930	0.000
	REE	0.067	0.082	0.042	1.931	0.054
ATT	PR	–0.142	–0.200	0.067	–2.986	0.003

### Analysis of mediating effects

Table 8 shows that in the total effect of INO→IT, the *p*-value was less than 0.05, and the bias-corrected confidence interval (CI) did not include 0 (CI of INO→IT = [–0.29,–0.126]). This evidence supported the existence of the total effect. In the indirect effect of INO→PR→IT, the *p*-value was also less than 0.05 and the bias-corrected confidence interval (CI) did not include 0 (CI of INO→PR→IT = [–0.191,–0.079]), so the hypothesis regarding the indirect effect was supported. The indirect effect of INO→PR→ATT→IT signified that the *p*-value was less than 0.05, *p*<0.05, and the bias-corrected CI did not include 0 (CI of INO→PR→ATT→IT = [–0.138,–0.019]), thus hypotheses related to the mediating effect was supported.

**TABLE 8:** Analysis of the indirect and total effects.

Effect	Regression weight	Standard error	*Z*-value	*P*-value	Lower bound of 95% CI	Upper bound of 95% CI
INO→PR→IT (Total indirect effect)	–0.199	0.042	–4.797	0.000	–0.29	–0.126
INO→PR→IT (Specific indirect effect)	–0.128	0.028	–4.525	0.000	–0.191	–0.079
INO→PR→ATT→IT (Specific indirect effect)	–0.071	0.03	–2.386	0.017	–0.138	–0.019

### Moderation effect

The subjective norm was a moderator of the proposed model. As shown in Table 9, the interaction of perceived risk and subjective norm to travel intention was 0.032 (*z* = | 0.791| <1.96, *p* = 0.429). Hence, subjective norms did not exist in the moderating effect.

**TABLE 9:** Effect of a moderator.

Endogenous variables	Exogenous variables	Estimate	Standard error	*Z*-value	*P*-value
IT	PR	–0.181	0.060	–3.023	0.002
	ATT	0.574	0.051	11.231	0.000
	REE	0.035	0.047	0.742	0.458
	SUN	0.364	0.058	6.313	0.000
	PR*SUN	0.032	0.040	0.791	0.429

## Conclusion and discussion

This study determines the effect of information overload, perceived risk, attitude, and response self-efficacy on tourists’ intention to travel in the post-COVID-19 era in Hainan, China. It also determines whether the subjective norm regulates tourists’ perceived risk and affects their intention to travel. Quantitative analysis of a questionnaire verifies the causal relationship between the research variables.

### The effect of information overload on perceived risk

In terms of a causal relationship, information overload was in a significant association with perceived risk, so the higher the perceived information overload, the greater would be the perceived risk. The development of COVID-19 and an excess of information have a significant impact on the average person. Excessive, serious, uncertain, and professional information have been prevalent since the COVID-19 pandemic, and because of the Internet, an overwhelming amount of information has increased the perception of risk.

The results of this study are similar to those of the study by Castañeda et al. (2020) and Abbasi et al. (2021). Modern society features an excessive amount of information, so it is difficult to make quick decisions. The information overload item scores for this study showed that the item with the highest score is “Since the COVID-19 pandemic, I feel that there is too much travel information.” The item with the lowest score was “Since the COVID-19 pandemic, I don’t know where to find the information I need.”

The results of this study showed that there is a significant increase in the perception of travel risk due to information overload. The plethora of COVID-19-related prevention and travel-related information means that a government should use propaganda and its credibility to reduce the deleterious effect of information overload. Various governments used the same accountability window to publish important messages through the media and to clearly convey correct instructions through simple procedures. This is common practice during this COVID-19 pandemic.

### The effect of perceived risk, attitude, and response self-efficacy on intention to travel

The impact of perceived risk on intention to travel was statistically significant. The greater the perceived risk, the lower the willingness to travel. These results were also realized by Rahmafitria et al. (2021). In the post-COVID-19 era, the perceived risk of tourism is an influencing factor for the intention to travel.

The regression coefficient for perceived risk to intention to travel was - 0.273, which was statistically significant. The greater the perceived risk, the lower the willingness to travel. These results were also realized by Rahmafitria et al. (2021) for the post-COVID-19 era. The perceived risk of tourism is an influencing factor for tourism willingness.

In terms of the scores for the perceived risk items, the item with the highest score is: “Since the new crown epidemic, tourism will increase uncertainty.” On the other hand, the item with the lowest score is: “Since the COVID-19 pandemic, I have not felt well protected.” Therefore, although uncertainty about travel has increased significantly, individuals have confidence in self-protection.

According to the findings of the research study, it can be implied that the attitude toward planning behavior theory is a factor that influences intention. The attitude of tourists is the decisive factor and determines tourists’ intention to travel. Hence forth, perceived risk had a significant impact on tourism attitude. The highest score for intention to travel was for “I would like to travel to the resort.” The lowest score was “I will encourage others to travel.” This shows that individuals are willing to travel but are unwilling to encourage others to travel. This result is similar to that of the effect of adjustment for this study.

The effect of response self-efficacy on intention to travel was not significant. For response self-efficacy, the highest score was for the item “I have the necessary knowledge for good COVID-19 measures in keeping me free of COVID-19 during the trips.” This shows that most individuals are confident in their knowledge of self-protection measures for the COVID-19 virus.

### Mediating and moderating effects

In terms of mediation effects, information overload had an indirect effect on the intention to travel through perceived risk. Information overload also had a chain-like mediation effect on the intention to travel through perceived risk and traveling attitude. Subjective norms define the importance of third-party opinions regarding traveling in the post-COVID-19 era. This study has different results from that by Rahmafitria et al. (2021). The subjective norms for this study did not play an important role. It is possible that travelers did not pay too much attention to other people’s perspectives.

## Theoretical contributions

This research has a variety of theoretical insights. The statistical result of the study validates the applicability of information overload, perceived risk, and TPB in affecting the tourists’ intention to travel. First, this research represents the investigator’s intent to the crucial moderating role of the subjective norm between perceived risk and intention to travel. This investigation aimed to uncover the post-expenditure interests of the tourists and investigate the gap between the proclaimed perceived risks and the real intention to travel. Second, by incorporating the extended TPB framework, the paper expands the present amount of research on the tourism industry. Third, the existing research encompasses the notion of information overload to the tourist intention to travel. This suggests the responsibility of information overload in the tourism business and its capability to affect the inner attitude of the tourists. Finally, the relationships investigated in this research deliver several prospects for tourism industry managers that would aid them to reconsider their current set of approaches to maintaining and drawing tourists.

## Practical implications

The research has several practical suggestions for corporate executives and practitioners. First, the recognition of information overload aspects that impact the tourist intention procedure will support the industries to reform their policies to improve tourists’ trust and improve their intention to travel, which can be achieved by placing several computer-generated checks on the internet. Second, current research demonstrates that the tourist is affected by extreme socialization, and its associated need for spontaneous reaction further enhances societal pressure (Zubair et al., 2022). Government administrators and hotel managers can limit the number of tourists at the tourist destination with the help of implementing virtual limits on the number of bookings every day. Third, the latest information technology research implies that information overload leads to adverse results (Zubair et al., 2022). Practitioners can restrict the information on the internet. Furthermore, as per the literature explained in this research study, it can be implied that tourists are more concerned regarding health procedures. Hence, the practitioners are required to guarantee that health-associated sanitary procedures are taken at tourist destinations.

## Research limitations and future research directions

The results of this study pertain only to travelers in Hainan Province, China. The COVID-19 pandemic is lengthy and there are many types of mutated viruses. Vaccines are being administered, but there is still a lot of unclear information. Information overload is an ongoing issue. Future studies are advised to focus on the individuals’ response to life after COVID-19, in terms of travel needs, entertainment, work, and schooling. Furthermore, future research studies can also focus to tackle the issue of information overload. Also, future verification studies will provide a reference for decision-making. Lastly, since this research study is conducted in China, which is an emerging economy, hence this study can be replicated in other emerging economies. Furthermore, future researchers can gain more insights by implementing the research model in developed economies. Finally, future researchers can also conduct a comparative analysis between emerging and developing economies.

## Data availability statement

The raw data supporting the conclusions of this article will be made available by the authors, without undue reservation.

## Ethics statement

Ethical review and approval was not required for the study on human participants in accordance with the local legislation and institutional requirements. Written informed consent from the patients/participants OR patients/participants legal guardian/next of kin was not required to participate in this study in accordance with the national legislation and the institutional requirements.

## Author contributions

HW, QC, J-MM, and H-LH: conceptualization. QC and J-MM: data curation and investigation. HW and H-LH: formal analysis. All authors read and agreed to the published version of the manuscript.
